# Feasibility of Adjuvant Radiotherapy or Chemoradiation for Elderly Patients with Squamous Cell Carcinoma of the Head and Neck, and Its Correlation with Different Comorbidity Scores: A Retrospective Cohort Study

**DOI:** 10.3390/cancers17142283

**Published:** 2025-07-09

**Authors:** Christoph Suess, Matthias Hipp, Tobias Ettl, Julian Kuenzel, Julia Maurer, Anna Ratzisberger, Fabian Baier, Felix Steger, Oliver Koelbl, Matthias Hautmann

**Affiliations:** 1 Klinik und Poliklinik für Strahlentherapie, Universitätsklinikum Regensburg, 93053 Regensburg, Germany; anna.rauscher@ra-rauscher.de (A.R.); f.baier@ukr.de (F.B.);; 2 Klinik für Strahlentherapie, Klinikum St. Marien Amberg, 92224 Amberg, Germany; 3 Klinik und Poliklinik für Mund-, Kiefer-und Gesichtschirurgie, Universitätsklinikum Regensburg, 93053 Regensburg, Germany; 4 Klinik und Poliklinik für Hals-Nasen-Ohren-Heilkunde, Universitätsklinikum Regensburg, 93053 Regensburg, Germany; julian.kuenzel@ukr.de; 5 UCC-R Universitäres Onkologisches Zentrum Regensburg, Franz-Josef-Strauß-Allee 11, 93053 Regensburg, Germany; 6 Klinik für Strahlentherapie und Radioonkologie, Klinikum Traunstein, 83278 Traunstein, Germany

**Keywords:** head and neck cancer, SCCHN, elderly patients, frail patients, radiotherapy, comorbidity score

## Abstract

**Background**: With aging populations, the incidence of squamous cell carcinoma of the head and neck (SCCHN) among elderly patients is increasing. Although adjuvant radiotherapy or chemoradiation is a well-established component of multimodal treatment, elderly patients remain underrepresented in clinical trials. This study evaluates the feasibility of adjuvant radiotherapy and chemoradiation in patients over 70 years with SCCHN and explores the correlation between treatment feasibility and various comorbidity scores. **Methods**: We retrospectively analyzed patients over 70 years of age who received adjuvant radiotherapy or chemoradiation at the University Hospital Regensburg between 2004 and 2018. A total of 71 patients, with a median age of 75 years, were included. The majority were classified as UICC stage IVa. Median follow-up was 27 months. **Results**: Sixty-two patients completed treatment without interruption, and sixty-five received at least 95% of the prescribed radiation dose. The median total dose was 64 Gy. Acute toxicity of grade III or IV (CTC) occurred in 37 patients. Local tumor control rates were 99% at 12 months, 88% at 24 months, and 76% at 5 years. Overall survival rates were 87% at 12 months, 67% at 24 months, and 41% at 60 months, with a median overall survival of 51 months. The Elixhauser Comorbidity Score showed significant predictive value for treatment feasibility (*p* = 0.006). **Conclusions**: Adjuvant radiotherapy and chemoradiation are feasible and effective treatment options for elderly patients with SCCHN. The favorable local and locoregional control rates reported here suggest, in line with other recent reports in the literature, that age alone should not be a justification for treatment de-intensification.

## Simple Summary

This study examined how well elderly patients with head and neck cancer tolerated radiation therapy or combined radiation and chemotherapy after surgery. We found that treatment outcomes were affected more by patients’ overall health and level of frailty than by age alone, while health scoring systems helped to predict tolerability. These findings suggest that older patients should not be denied standard cancer treatment just because of their age.

## 1. Introduction

Squamous cell carcinoma of the head and neck (SCCHN) is among the most common malignancies worldwide, affecting populations in both developed and developing countries. In 2018, the global incidence of SCCHN was estimated to be 834,860 cases, accounting for 4.6% of all newly diagnosed cancers [1]. For instance, in the United States in 2019, approximately 53,000 new cases of oral cavity and pharyngeal cancers, and 12,410 cases of laryngeal cancer, were reported, representing about 3.7% of all new cancer diagnoses in the country [2]. In Germany, cancer statistics are published biennially by the Robert Koch Institute (RKI) in collaboration with the Society of Epidemiological Cancer Registries in Germany. In 2020, 14,220 new cases of tumors in the oral cavity and pharynx (9730 in men and 4490 in women), along with 3540 new cases of laryngeal carcinoma (2990 in men and 550 in women), were recorded [3].

The etiology of SCCHN is multifactorial. Key risk factors include tobacco use, excessive alcohol consumption, infection with human papillomavirus (HPV), and various premalignant conditions, whether inherited or acquired (e.g., immunodeficiency) [4,5,6]. SCCHN is a heterogeneous disease, often diagnosed at a locally advanced stage [7,8]. In Europe, a multimodal treatment approach is commonly employed, typically involving surgical tumor resection followed by adjuvant therapy [9,10,11,12]. According to both national and international guidelines, the choice between adjuvant radiotherapy and chemoradiation is based on TNM staging, specific risk factors, general health status, and the presence of comorbidities [10,11,12,13,14]. As populations age, the number of elderly patients diagnosed with SCCHN is increasing, presenting new challenges for radiation oncologists, who now more frequently encounter older and/or frail patients [15,16,17]. The concept of frailty has been discussed in the literature [18], and screening tools have been developed to assess it [19,20,21,22,23]. Similarly, several comorbidity scoring systems exist to help evaluate treatment suitability in elderly patients, including the Karnofsky Performance Status, Charlson Comorbidity Index, and Elixhauser Comorbidity Score [22,24,25,26,27,28,29]. Despite these tools, elderly or frail patients remain underrepresented in clinical trials, and real-world data on the feasibility and outcomes of adjuvant radiotherapy or chemoradiation in this population are limited [18,30,31,32,33].

The aim of this study was to evaluate the feasibility and outcomes of adjuvant radiotherapy or chemoradiation in a real-world cohort of patients aged 70 years and older with SCCHN. Furthermore, we assessed the predictive value of various comorbidity scores—specifically, the Karnofsky Performance Status, the Charlson Comorbidity Index, and the Elixhauser Comorbidity Score—in relation to treatment feasibility and survival outcomes.

## 2. Materials and Methods

This retrospective study included patients aged 70 years or older with histologically confirmed squamous cell carcinoma of the head and neck (SCCHN) who received adjuvant radiotherapy or chemoradiation. All patients were treated at the University Hospital Regensburg between 2004 and 2018. The study was conducted in accordance with the Declaration of Helsinki and received approval from the Ethics Committee of the University of Regensburg.

**Figure A1:**
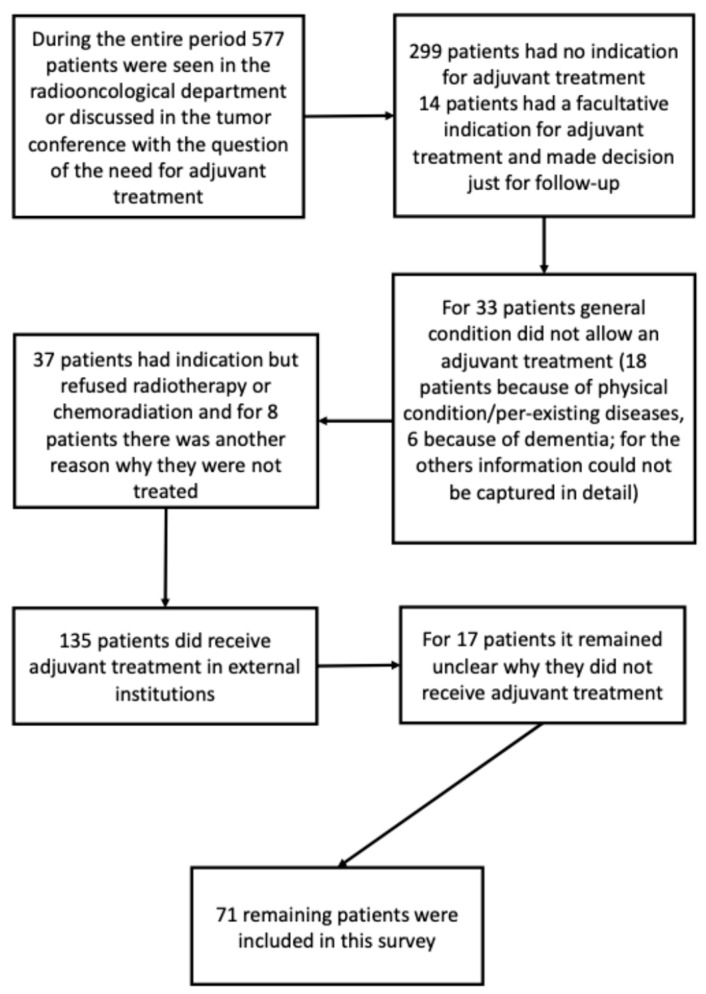
Consort diagram.

During the study period, 577 elderly patients with SCCHN were evaluated in the Department of Radiation Oncology or presented at the institutional tumor board for assessment of indication for adjuvant therapy. Among these, 299 patients were not eligible for adjuvant radiotherapy or chemoradiation due to tumor stage and/or absence of relevant risk factors. Detailed reasons for non-inclusion are shown in Figure A1. Ultimately, 71 patients (16 women and 55 men) were included in the final analysis. The median age was 75 years (interquartile range [IQR]: 72–79 years). Forty-two patients were classified as UICC stage IVa. In 20 patients, adjuvant treatment was administered following resection of tumor recurrence; however, none had previously received head-and-neck radiotherapy. Sixty-two patients underwent adjuvant radiotherapy alone, and nine received concomitant chemoradiation. The median follow-up was 27 months (IQR: 18–72 months) (Table 1). The concurrent chemotherapy consisted of platinum-based protocols (Cisplatin, Carboplatin, and Cisplatin/5-FU), with the most frequently used regimen being weekly cisplatin at 40 mg/m^2^ with a planned dose of 200 mg/m^2^ corresponding to five cycles. In this cohort we used conventional and hyperfractionated accelerated radiotherapy in 93% and 7% of cases, respectively. All radiation treatments were performed with a linear accelerator, using 3D-conformal radiation therapy in nine patients (12.7%) and IMRT or VMAT in sixty-two patients (87.3%).

For this study, tumors located in the oral cavity, oropharynx, larynx, and hypopharynx were collectively categorized as SCCHN. The primary aim was to assess the feasibility of adjuvant radiotherapy or chemoradiation in elderly patients. Comorbidity was evaluated using the Karnofsky Performance Status (KPS), the Charlson Comorbidity Index (CCI), and the Elixhauser Comorbidity Score (ECS), based on documented secondary diagnoses and preexisting conditions (Table 2). Notably, the Charlson Comorbidity Index was calculated without age adjustment to allow better differentiation within this elderly cohort. The CCI encompasses 17 factors, while the ECS includes 30 parameters.

Treatment feasibility and outcomes were analyzed in relation to comorbidity scores using binary logistic regression. Clinical data were extracted from institutional documentation systems, including Mosaiq (IMPAC Medical Systems, Elekta, Stockholm, Sweden), SAP (SAP, Walldorf, Germany), Onkodat (MedicDAT GmbH, Poikam, Germany), and CATO (Becton Dickinson, Franklin Lakes, NJ, USA). Survival data were completed using information from the local registration office and the Tumor Centre Regensburg.

Acute and late toxicities were documented during treatment and at scheduled follow-up visits (6 weeks, 3-, 6-, and 12-months post-radiotherapy). Adverse events were graded using the Common Terminology Criteria for Adverse Events (CTCAE) Version 3.0, in line with the documentation practices prevailing at the time [34].

Statistical analyses were performed using IBM SPSS Statistics Version 25 (IBM, Armonk, NY, USA). A *p*-value < 0.05 was considered statistically significant. Depending on variable type, t-tests were applied for continuous variables, Mann–Whitney U tests for ordinal variables, and Chi-squared tests for categorical variables. Kaplan–Meier survival curves were generated for up to 60 months and compared using the log-rank test. Predictive factors for treatment feasibility and overall survival were identified using Cox proportional hazards regression modeling in a two-step approach. Initially, a univariate Cox regression analysis was performed to evaluate the association of individual clinical and demographic variables with treatment feasibility and survival outcomes. Variables demonstrating a *p*-value < 0.10 in the univariate analysis were considered for inclusion in the subsequent multivariate model to avoid premature exclusion of potentially relevant factors. In the second step, a multivariate Cox proportional hazards regression was conducted to identify independent predictors while controlling for confounding effects. A *p*-value < 0.05 was considered statistically significant in the final multivariate analysis.

## 3. Results

### 3.1. Feasibility

Adjuvant radiotherapy and chemoradiation were generally well tolerated among the elderly patients in this cohort. Of the 71 patients included, 62 (87.3%) completed the planned treatment without interruption. In only nine patients (12.7%) was treatment interrupted for two or more days. A total of 65 patients (91.5%) received at least 95% of the prescribed radiation dose. Six patients (8.5%) received less than 95% of the dose, including four patients (5.6%) who received less than 80%. The median radiation dose was 64 Gy (interquartile range [IQR]: 60–66 Gy).

Nine patients (12.7%) received concomitant chemoradiation. Among these, six (66.7%) received at least 75%, and eight (88.9%) received at least 50% of the planned chemotherapy dose. One patient received less than 25% of the intended chemotherapy due to early treatment termination. Several chemotherapy cycles were postponed due to bone marrow suppression, including leukopenia and/or neutropenia.

Binary logistic regression revealed that the Elixhauser Comorbidity Score significantly predicted treatment feasibility, specifically the ability to receive ≥95% of the prescribed radiation dose (*p* = 0.006). Neither the Karnofsky Performance Status (KPS) nor the Charlson Comorbidity Index (CCI) showed statistically significant predictive value in this context.

### 3.2. Acute Toxicity

Acute toxicities were evaluated, including oral mucositis, dysphagia, radiation dermatitis, xerostomia, weight loss, renal dysfunction, and hematologic changes such as leukopenia, anemia, and thrombocytopenia. Grade III acute toxicity occurred in 37 patients (52.1%). Chi-squared testing showed no significant increase in grade III toxicities among patients receiving chemoradiation compared to those receiving radiotherapy alone. The most common severe toxicity was grade III oral mucositis, reported in twenty-four patients (33.8%), with three patients (4.2%) still experiencing symptoms six weeks post-treatment. Grade III dysphagia occurred in twenty-seven patients (38.0%) during therapy, and persisted in nine patients (12.7%) at six weeks. Ten patients (14.1%) had pre-existing dysphagia prior to treatment, which was not classified as treatment-related unless worsened. Four patients (5.6%) lost more than 10% of their body weight during therapy. Grade III radiation dermatitis was observed in 10 patients (14.1%).

### 3.3. Late Toxicity

Persistent grade III dysphagia beyond 6 and 12 months was recorded in four (5.6%) and three (4.2%) patients, respectively. Xerostomia grade III was present in four patients (5.6%) at six weeks post-treatment, and remained in two (2.8%) at six months, and in one (1.4%) at 12 months. The Karnofsky Performance Status remained stable in most patients, with a decline of at least 20% observed in only five patients (7.0%) during follow-up.

### 3.4. Survival and Treatment Response

**Figure 1:**
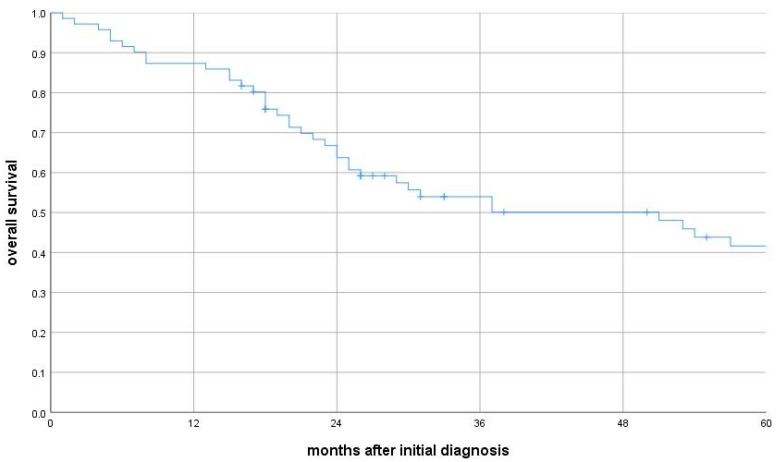
Overall survival in the whole cohort.

All patients were treated with curative intent. The overall survival (OS) rates were 87% at 12 months, 67% at 24 months, and 41% at 60 months (Figure 1). Median OS was 51 months (IQR: 19–99 months). Gender and age had no significant impact on survival outcomes. Median OS by age group was as follows:

• 70–74 years: 37 months (n = 34)
• 75–79 years: 54 months (n = 24)
• 80–84 years: 51 months (n = 10)
• ≥85 years: 24 months (n = 3)

**Figure 2:**
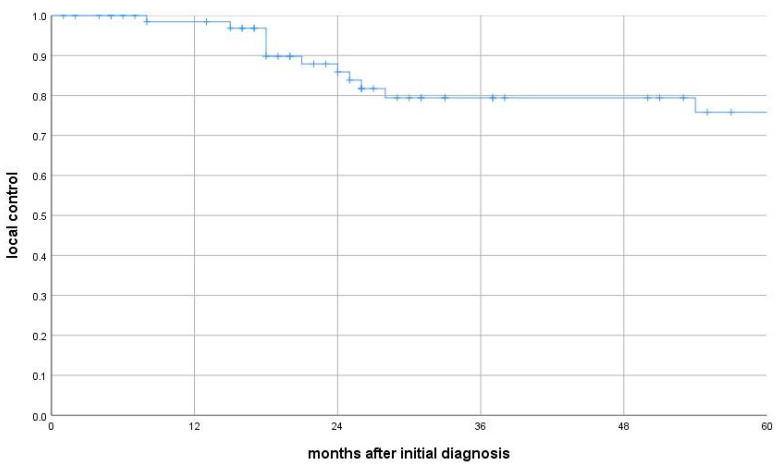
Local tumor control after adjuvant radiotherapy/chemoradiation.

Seventeen patients (23.9%) experienced local, locoregional, or metastatic recurrence, with a median time to recurrence of 12 months (IQR: 10–21.5 months). Thirteen of these patients developed metastatic disease (median 11 months, IQR: 9–20). Median relapse-free survival was 25 months (IQR: 12–57), local-relapse-free survival 25 months (IQR: 15–65), and metastasis-free survival 26 months (IQR: 14–65). Median OS for patients with tumor recurrence was 21 months (IQR: 18–54), significantly shorter than for those without recurrence (65 months; IQR: 23–120; log-rank test: *p* = 0.001). Local control was 99% at 12 months, 88% at 24 months, and 76% at 60 months. Thirteen patients had locoregional recurrence (median time: 25 months; IQR: 15–65) (Figure 2). Subgroup analysis revealed that median OS for patients with local recurrence and those with metastatic spread was 24 months (IQRs: 18–54 and 14–25 months, respectively), both significantly shorter than among recurrence-free patients (local relapse: *p* = 0.039; metastasis: *p* = 0.002).

### 3.5. Univariate and Multivariate Analysis of Prognostic Factors

Univariate analysis revealed significantly reduced OS among patients with a KPS ≤ 60% before, during, or after treatment (*p* = 0.019), with KPS ≤ 50% after radiotherapy showing particularly poor prognosis (KPS 30%: *p* = 0.000; KPS 40%: *p* = 0.000; KPS 50%: *p* = 0.001). A lower CCI (indicating a lower comorbidity burden) before therapy was associated with improved survival (*p* = 0.023). Tumor recurrence and metastatic spread during follow-up were both significantly associated with reduced OS (*p* = 0.002 and *p* = 0.001, respectively). Dysphagia grade III six months after treatment showed a trend toward worse survival (*p* = 0.071). Other non-significant trends included anemia grade III before treatment (*p* = 0.067), tumor recurrence at the time of radiation initiation (*p* = 0.101), and UICC stage IVb (*p* = 0.084). Multivariate analysis identified two independent predictors of worse overall survival: tumor recurrence (local or metastatic) during follow-up (*p* = 0.02) and persistent grade III dysphagia six months post-treatment (*p* = 0.036).

## 4. Discussion

In this survey, we consciously decided to focus on elderly patients aged over 70 years with SCCHN who had been treated with adjuvant radiotherapy or chemoradiation at the University Hospital Regensburg. Our aim was to investigate this real-world patient cohort to assess the reality and authenticity of the therapy. We sought also to analyze how these patients tolerated treatment and how best to manage the demands and challenges of this specific subgroup of patients with head and neck cancer. The majority of other studies have focused on pre-selected patient groups. Such (pre-)selection may have biased the results of surveys and overlooked the specific requirements of treating elderly or frail patients—one reason for the current lack of sufficient and valid real-world data in this population.

For elderly patients with locally advanced head and neck cancer, radiotherapy and/or chemoradiation are important and effective treatment options, particularly in the adjuvant setting [10]. Opinions differ regarding the de-escalation of adjuvant therapy. Some study groups argue that elderly patients should not be excluded from radiotherapy or chemoradiation solely based on age [30,35,36]. We support this view, based on the good feasibility of radiotherapy and chemoradiation observed in our patient cohort. Elderly and frail patients appear to benefit from these treatments similarly to younger patients with head and neck cancer. Nevertheless, careful patient selection with respect to comorbidities and Karnofsky performance status is necessary [37]. Future prospective studies on radiotherapy and chemoradiation in elderly patients with head and neck cancer should focus particularly on frail patients.

It is necessary to examine our subgroup of elderly SCCHN patients more closely. The main reasons for interruptions in radiotherapy were acute toxicity or other medical complications, such as infections. Only a few interruptions resulted from patient non-compliance or organizational issues. Nonetheless, 65 patients were able to complete radiotherapy with 95% of the initially prescribed radiation dose (91.5%). Four patients (5.6%) received less than 80% of the prescribed dose due to premature treatment termination. One of these patients died of respiratory insufficiency due to rapidly progressing pulmonary metastases newly diagnosed during radiotherapy (16 Gy). Another patient died of cardiovascular disease during chemoradiation (16.2 Gy and less than 25% of the prescribed chemotherapeutic dose). Two patients discontinued radiotherapy at their own request.

Elderly patients are often denied radiotherapy or chemoradiation solely based on age. Metges et al. observed that elderly patients were frequently treated less aggressively due to assumptions about limited life expectancy. In their survey, Metges et al. found no differences in treatment response among various age groups [30], a finding that is consistent with our results. Straube and Pigorsch et al. also reported good radiotherapy outcomes in elderly and frail patients [38]; however, their patient cohort was smaller than ours. We also found no evidence to support the hypothesis that elderly patients generally have a worse prognosis compared to younger or differently aged cohorts. Most of our patients had locally advanced tumors (UICC stage IVa), accounting for the poor overall survival rate.

Physicians often presume that the treatment process would impair quality of life. However, elderly patients have been shown to demonstrate comparable performance regarding quality of life when compared with general patient populations [39]. The implementation of intensity-modulated radiotherapy—with its associated reduction in dosage leading to improved protection of organs at risk—has improved the tolerability of radiotherapy in the head and neck region [35].

Comparing subgroups in terms of whether chemoradiation or radiotherapy should be applied is difficult, as postoperative patients with intermediate risk usually receive radiotherapy alone [10]. According to current guidelines, nearly all high-risk patients in our cohort would have been recommended to receive simultaneous chemoradiation had they been younger. In practice, only the fit elderly patients in our cohort received chemoradiation, because many frail patients were not expected to tolerate it. These decisions were made in line with the data from the “Meta-analysis of Chemotherapy in Head and Neck Cancer (MACH-NC)” [40]. Patients in our cohort with high-risk factors (ECS, more than three affected lymph nodes, margins < 5 mm), but poor comorbidity scores, received radiotherapy alone—even though guidelines would have recommended chemoradiation [10,40]. However, some elderly patients with high-risk features received simultaneous chemoradiation due to their good general condition and Karnofsky performance status, despite the MACH-NC recommending radiotherapy alone for patients over 70. This was also considered in light of prospective randomized trials that included patients up to 75 years of age [31,32]. In our study, chemoradiation was not significantly less well tolerated by elderly patients compared to younger ones—a finding consistent with results of other surveys [41,42,43].

Patients treated with chemoradiation in our study tended to have shorter survival than those treated with radiotherapy alone. This is likely due to the fact that patients receiving chemotherapy had significant risk factors (ECS, multiple affected lymph nodes, close margins) and therefore faced a higher risk of recurrence and reduced survival. Additionally, it must be acknowledged that patients with such risk factors who did not receive chemotherapy due to frailty or comorbidities may already have had a limited life expectancy. As such, comparing and interpreting these patient groups remains challenging. We believe that treatment decisions should be individualized, particularly for patients between the ages of 70 and 75.

Our multivariate analysis (Cox regression) showed a significant association between dysphagia—especially at 6 months—and overall survival. Dysphagia may be related to tumor burden at the start of therapy, or it may be therapy-induced. Adequate nutritional support could prevent weight-loss and deterioration in general condition. Dysphagia and swallowing impairment are complex clinical issues. Detection may be difficult in small patient cohorts. Because aspiration pneumonia is a common complication, access to phoniatric and logopedic care is essential [44,45,46,47]. Therefore, treatment in specialized centers is desirable. The still-active field of research on radiosensitizers, radioenhancers, and radioprotective strategies might be an additional tool to further improve the tolerability of radiation therapy, especially in this cohort of patients [48]. All patients in this survey were treated at the University Hospital Regensburg for the entire duration of therapy. Our real-world patient cohort was larger and less heterogeneous than those in other studies. Each patient received adjuvant radiotherapy or chemoradiation, and it appears that the cohort was representative of clinical reality. Any suspicion that this was a pre-selected cohort can be refuted, especially given that only 33 out of an initial 278 patients with a general indication for adjuvant therapy were not fit for treatment.

## 5. Conclusions

Squamous cell carcinomas of the head and neck are common malignant tumors; however, elderly patients have been underrepresented in scientific analyses to date. The aim of the present study was to evaluate the feasibility and outcomes of radiotherapy or chemoradiation in patients aged over 70 years with head and neck tumors, and to correlate these outcomes with various frailty scores.

The feasibility of radiotherapy and chemoradiation in our cohort was good, with local tumor control being particularly satisfactory. Careful patient selection based on comorbidities, along with the use of established comorbidity scores such as the Karnofsky Performance Status, Charlson Comorbidity Score, and Elixhauser Comorbidity Score, appears to be advisable. The Elixhauser and Charlson scores can offer valuable guidance in assessing an elderly patient’s suitability for radiation therapy prior to treatment initiation.

Age alone does not appear to significantly influence overall survival, as no clear difference was observed between elderly patients and younger or unselected patient populations. Our findings suggest that de-intensification of treatment based solely on age should be critically re-evaluated. Further research is necessary to more accurately assess cancer treatment strategies, particularly in elderly and frail patients.

## Figures and Tables

**Table 1:** Patient characteristics.

Patient Characteristics		Percentage
Patients [n]	71	
Gender [n]		
• Female	16	22.5%
• Male	55	77.5%
Age [years] at diagnosis		
• Median	74	
• First quartile	71	
• Third quartile	78	
Tumor entity [n]		
• Oropharynx	19	26.7%
• Oral cavity	29	40.8%
• Hypopharynx	7	9.9%
• Larynx	16	22.5%
UICC classification [n]		
I	5	7.0%
II	3	4.2%
III	18	25.4%
Iva	42	59.4%
IVb	3	4.2%
Initial Karnofsky performance status [n]		
30–40%	2	2.8%
50–60%	12	16.9%
70–80%	42	59.2%
90–100%	10	14.1%
Not specified	5	7.0%
Minimum Karnofsky performance status during therapy [n]		
30–40%	3	4.2%
50–60%	21	29.5%
70–80%	35	49.3%
90–100%	4	5.6%
Not specified	8	11.3%

**Table 2:** Comorbidity Scores.

Comorbidity Scores		Percentage
Patients [n]	71	Data
Charlson comorbidity score initially [n]		
1–2	10	14.1%
3–4	39	54.9%
5–6	18	25.4%
7–11	4	5.6%
Elixhauser comorbidity score initially [n]		
(−1)–5	15	21.1%
6–10	14	19.7%
11–15	22	31.0%
16–20	8	11.3%
21–25	7	9.9%
26–30	5	7.0%

## Data Availability

The raw data supporting the conclusions of this article will be made available by the authors on request.

## Abbreviations

The following abbreviations are used in this manuscript:

CCI	Charlson Comorbidity Index
CTC	Common toxicity criteria
ECS	Elixhauser Comorbidity Score
HPV	Human papillomavirus
KPS	Karnofsky Performance Index
OS	Overall survival
SCCHN	Squamous cell carcinoma of the head and neck

## References

[1] Bray F., Ferlay J., Soerjomataram I., Siegel R., Torre L., Jemal A. (2018). Global cancer statistics 2018: GLOBOCAN estimates of incidence and mortality worldwide for 36 cancers in 185 countries. CA Cancer J. Clin..

[2] Siegel R.L., Miller K.D., Jemal A. (2019). Cancer statistics, 2019. CA Cancer J. Clin..

[3] Robert Koch-Institut, und die Gesellschaft, der epidemiologischen, Krebsregister in Deutschland e.V. (2023). Krebs in Deutschland Für 2019/2020.

[4] Shaw R., Beasley N. (2016). Aetiology and risk factors for head and neck cancer: United Kingdom National Multidisciplinary Guidelines. J. Laryngol. Otol..

[5] Blot W.J., McLaughlin J.K., Winn D.M., Austin D.F., Greenberg R.S., Preston-Martin S., Bernstein L., Schoenberg J.B., Stemhagen A., Fraumeni J.F. (1988). Smoking and drinking in relation to oral and pharyngeal cancer. Cancer Res..

[6] Sankaranarayanan R., Masuyer E., Swaminathan R., Ferlay J., Whelan S. (1998). Head and neck cancer: A global perspective on epidemiology and prognosis. Anticancer Res..

[7] Magnes T., Egle A., Greil R., Melchardt T. (2017). Update on squamous cell carcinoma of the head and neck: ASCO annual meeting 2017. Memo.

[8] Marur S., Forastiere A.A. (2016). Head and Neck Squamous Cell Carcinoma: Update on Epidemiology, Diagnosis, and Treatment. Mayo Clin. Proc..

[9] Haussmann J., Tamaskovics B., Bölke E., Djiepmo-Njanang F.J., Kammers K., Corradini S., Hautmann M., Ghadjar P., Maas K., Schuler P.J. (2019). Addition of chemotherapy to hyperfractionated radiotherapy in advanced head and neck cancer-a meta-analysis. Strahlenther. Onkol..

[10] National Comprehensive Cancer Network (2025) NCCN Guidelines Head and Neck Cancers, National Comprehensive Cancer Network. Head and Neck Cancers (Version 2.2025). https://www.nccn.org/professionals/physician_gls/pdf/head-and-neck.pdf.

[11] Bernier J., Domenge C., Ozsahin M., Matuszewska K., Lefèbvre J.L., Greiner R.H., Giralt J., Maingon P., Rolland F., Bolla M. (2004). Postoperative irradiation with or without concomitant chemotherapy for locally advanced head and neck cancer. N. Engl. J Med..

[12] Bernier J., Cooper J.S., Pajak T.F., Glabbeke M., Bourhis J., Forastiere A., Ozsahin E.M., Jacobs J., Jassem J., Ang K.K. (2005). Defining risk levels in locally advanced head and neck cancers: A comparative analysis of concurrent postoperative radiation plus chemotherapy trials of the EORTC (#22931) and RTOG (# 9501). Head Neck.

[13] Gregoire V., Lefebvre J.-L., Licitra L., Felip E. (2010). Squamous cell carcinoma of the head and neck: EHNS-ESMO-ESTRO Clinical Practice Guidelines for diagnosis, treatment and follow-up. Ann. Oncol..

[14] Machiels J.P., Leemans C.R., Golusinski W., Grau C., Licitra L., Gregoire V. (2020). Squamous cell carcinoma of the oral cavity, larynx, oropharynx and hypopharynx: EHNS–ESMO–ESTRO Clinical Practice Guidelines for diagnosis, treatment and follow-up. Ann. Oncol..

[15] Sikora A.G., Toniolo P., DeLacure M.D. (2004). The changing demographics of head and neck squamous cell carcinoma in the United States. Laryngoscope.

[16] Jelinek M.J., Howard A.S., Haraf D.J., Vokes E.E. (2018). Management of Early Head and Neck Cancer in Elderly Patients. JCO Oncol. Pract..

[17] VanderWalde N.A., Fleming M., Weiss J., Chera B.S. (2013). Treatment of Older Patients with Head and Neck Cancer: A Review. Oncologist.

[18] Clegg A., Young J., Iliffe S., Rikkert M.O., Rockwood K. (2013). Frailty in elderly people. Lancet.

[19] National Comprehensive Cancer Network (2019). NCCN Clinical Practice Guidelines in Oncology: Older Adult Oncology. https://www.nccn.org/professionals/physician_gls/default.aspx#site.

[20] Pottel L., Boterberg T., Pottel H., Goethals L., Noortgate N.V.D., Duprez F., Neve W.D., Rottey S., Geldhof K., Eygen K.V. (2012). Determination of an adequate screening tool for identification of vulnerable elderly head and neck cancer patients treated with radio(chemo)therapy. J. Geriatr. Oncol..

[21] Biganzoli L., Boni L., Becheri D., Zafarana E., Biagioni C., Cappadona S., Bianchini E., Oakman C., Magnolfi S.U., Leo A.D. (2013). Evaluation of the cardiovascular health study (CHS) instrument and the Vulnerable Elders Survey-13 (VES-13) in elderly cancer patients. Are we still missing the right screening tool?. Ann. Oncol..

[22] Elixhauser A., Steiner C., Harris D.R., Coffey R.M. (1998). Comorbidity measures for use with administrative data. Med. Care.

[23] Bellera C.A., Rainfray M., Mathoulin-Pelissier S., Mertens C., Delva F., Fonck M., Soubeyran P.L. (2012). Screening older cancer patients: First evaluation of the G-8 geriatric screening tool. Ann. Oncol..

[24] Charlson M.E., Pompei P., Ales K.L., MacKenzie C.R. (1987). A new method of classifying prognostic comorbidity in longitudinal studies: Development and validation. J. Chronic Dis..

[25] Sundararajan V., Henderson T., Perry C., Muggivan A., Quan H., Ghali W.A. (2004). New ICD-10 version of the Charlson comorbidity index predicted in-hospital mortality. J. Clin. Epidemiol..

[26] Poses R.M., McClish D.K., Smith W.R., Bekes C., Scott W.E. (1996). Prediction of survival of critically ill patients by admission comorbidity. J. Clin. Epidemiol..

[27] Charlson M.E., Charlson R.E., Peterson J.C., Marinopoulos S.S., Briggs W.M., Hollenberg J.P. (2008). The Charlson comorbidity index is adapted to predict costs of chronic disease in primary care patients. J. Clin. Epidemiol..

[28] Quan H., Li B., Couris C.M., Fushimi K., Graham P., Hider P., Januel J.M., Sundararajan V. (2011). Updating and validating the Charlson comorbidity index and score for risk adjustment in hospital discharge abstracts using data from 6 countries. Am. J. Epidemiol..

[29] Oken M.M., Creech R.H., Tormey D.C., Horton J., Davis T.E., McFadden E.T., Carbone P.P. (1982). Toxicity and response criteria of the Eastern Cooperative Oncology Group. Am. J. Clin. Oncol..

[30] Metges J.P., Eschwege F., Crevoisier R., Lusinchi A., Bourhis J., Wibault P. (2000). Radiotherapy in head and neck cancer in the elderly: A challenge. Crit. Rev. Oncol. Hematol..

[31] Dietz A., Wichmann G., Kuhnt T., Pfreundner L., Hagen R., Schleich M., Kölbl O., Hautmann M.G., Strutz J., Schreiber F. (2018). Induction chemotherapy (IC) followed by radiotherapy (RT) versus cetuximab plus IC and RT in advanced laryngeal/hypopharyngeal cancer resectable only by total laryngectomy-final results of the larynx organ preservation trial DeLOS-II. Ann. Oncol..

[32] Fietkau R., Hecht M., Hofner B., Lubgan D., Iro H., Gefeller O., Rödel C., Hautmann M.G., Kölbl O., Salay A. (2020). Randomized phase-III-trial of concurrent chemoradiation for locally advanced head and neck cancer comparing dose reduced radiotherapy with paclitaxel/cisplatin to standard radiotherapy with fluorouracil/cisplatin: The PacCis-trial. Radiother. Oncol..

[33] Kuhnt T., Schreiber A., Pirnasch A., Hautmann M.G., Hass P., Sieker F.P., Engenhart-Cabilic R., Richter M., Dellas K., Dunst J. (2017). Hyperfractionated accelerated radiation therapy plus cetuximab plus cisplatin chemotherapy in locally advanced inoperable squamous cell carcinoma of the head and neck: Final 5year results of a phase II study (Hyperfraktionierte akzelerierte Bestrahlung plus Cetuximab plus Cisplatin-Chemotherapie beim lokal fortgeschrittenen, inoperablen Plattenepithelkarzinom im Kopf-Hals-Bereich: 5-Jahres-Ergebnisse einer Phase-II-Studie). Strahlenther. Onkol..

[34] National Cancer Institute Division of Cancer Treatment and Diagnosis Common Terminology Criteria for Adverse Events (CTCAE)|Protocol Development|CTEP. https://ctep.cancer.gov/protocolDevelopment/electronic_applications/ctc.htm#ctc_50.

[35] Kunkler I.H., Audisio R., Belkacemi Y., Betz M., Gore E., Hoffe S., Kirova Y., Koper P., Lagrange J.L., Markouizou A. (2014). Review of current best practice and priorities for research in radiation oncology for elderly patients with cancer: The International Society of Geriatric Oncology (SIOG) task force. Ann. Oncol..

[36] Wasil T., Lichtman S.M., Gupta V., Rush S. (2000). Radiation therapy in cancer patients 80 years of age and older. Am. J. Clin. Oncol..

[37] Sanabria A., Carvalho A.L., Vartanian J.G., Magrin J., Ikeda M.K., Kowalski L.P. (2007). Comorbidity is a prognostic factor in elderly patients with head and neck cancer. Ann. Surg. Oncol..

[38] Straube C., Pigorsch S.U., Scherb H., Wilkens J.J., Bier H., Combs S.E. (2016). Reduced volume SIB-IMRT/IGRT to head and neck cancer in elderly and frail patients: Outcome and toxicity. Radiat. Oncol..

[39] Rühle A., Haehl E., Kalckreuth T., Stoian R., Spohn S.K.B., Sprave T., Zamboglou C., Gkika E., Knopf A., Grosu A.L. (2021). Surviving Elderly Patients with Head-and-Neck Squamous Cell Carcinoma—What Is the Long-Term Quality of Life after Curative Radiotherapy?. Cancers.

[40] Blanchard P., Landais C., Petit C., Zhang Q., Grégoire V., Burtness B., Ghi M.G., Janot F., Overgaard J., Wolf G. (2016). Meta-analysis of chemotherapy in head and neck cancer (MACH-NC): An update on 100 randomized trials and 19,248 patients, on behalf of MACH-NC group. Ann. Oncol..

[41] Giovanazzi-Bannon S., Rademaker A., Lai G., Benson A.B. (1994). Treatment tolerance of elderly cancer patients entered onto phase II clinical trials: An Illinois Cancer Center study. J. Clin. Oncol..

[42] Lichtman S.M., Wildiers H., Chatelut E., Steer C., Budman D., Morrison V.A., Tranchand B., Shapira I., Aapro M. (2007). International Society of Geriatric Oncology Chemotherapy Taskforce: Evaluation of chemotherapy in older patients—An analysis of the medical literature. J. Clin. Oncol..

[43] Newcomb P.A., Carbone P.P. (1993). Cancer treatment and age: Patient perspectives. J. Natl. Cancer Inst..

[44] Patterson J.M. (2019). Late Effects of Organ Preservation Treatment on Swallowing and Voice; Presentation, Assessment, and Screening. Front. Oncol..

[45] Pedersen A., Wilson J., McColl E., Carding P., Patterson J. (2016). Swallowing outcome measures in head and neck cancer—How do they compare?. Oral Oncol..

[46] Brodsky M.B., Suiter D.M., Gonzalez-Fernandez M., Michtalik H.J., Frymark T.B., Venediktov R., Schooling T. (2016). Screening Accuracy for Aspiration Using Bedside Water Swallow Tests: A Systematic Review and Meta-Analysis. United States. Chest.

[47] Hey C., Lange B.P., Eberle S., Zaretsky Y., Sader R., Stöver T., Wagenblast J. (2013). Water swallow screening test for patients after surgery for head and neck cancer: Early identification of dysphagia, aspiration and limitations of oral intake. Anticancer Res..

[48] Ganau M., Foroni R.I., Gerosa M., Ricciardi G.K., Longhi M., Nicolato A. (2015). Radiosurgical options in neuro-oncology: A review on current tenets and future opportunities. Part II: Adjuvant radiobiological tools. Tumori.

